# Improving skeleton algorithm for helping *Caenorhabditis elegans* trackers

**DOI:** 10.1038/s41598-020-79430-8

**Published:** 2020-12-17

**Authors:** Pablo E. Layana Castro, Joan Carles Puchalt, Antonio-José Sánchez-Salmerón

**Affiliations:** Instituto de Automática e Informática Industrial, Universitat Politècnica de València, Valencia, Spain

**Keywords:** Imaging and sensing, Software

## Abstract

One of the main problems when monitoring *Caenorhabditis elegans* nematodes (*C. elegans*) is tracking their poses by automatic computer vision systems. This is a challenge given the marked flexibility that their bodies present and the different poses that can be performed during their behaviour individually, which become even more complicated when worms aggregate with others while moving. This work proposes a simple solution by combining some computer vision techniques to help to determine certain worm poses and to identify each one during aggregation or in coiled shapes. This new method is based on the distance transformation function to obtain better worm skeletons. Experiments were performed with 205 plates, each with 10, 15, 30, 60 or 100 worms, which totals 100,000 worm poses approximately. A comparison of the proposed method was made to a classic skeletonisation method to find that 2196 problematic poses had improved by between 22% and 1% on average in the pose predictions of each worm.

## Introduction

*Caenorhabditis elegans* is a very important free-living transparent nematode that has been used since 1960 as a development model in the biology field. Monitoring these worms allows behavioural data to be acquired in their life cycles, especially at advanced adult age when they present some disorders, physical deterioration, tissue deterioration and mobility, among other characteristics, similar to humans at advanced ages^1,2^. This makes it an ideal model organism for studying and treating several types of diseases^3^.

While investigating *C. elegans* behaviour, some advanced tools and techniques were developed to recognise the outward configurations, poses and coiled shapes that these nematodes can take when they roll up or aggregate with others while moving. Many of these systems require one end or both ends of *C. elegans* to be visible in order to identify and estimate a pose with different parameters, such as length^4^, smoothness^5,6^, previous segmentation^7^, or other complex models^8–13^.

Pose estimation models have been developed using neural networks, such as^14–16^, which have been shown to have made accurate predictions in their datasets. These models solve the problems of finding individual worm poses, such as coiled shapes or even self-occluded, although these tools pose a formidable challenge because the learned network is difficult to interpret. This involves resorting to highly trained people to understand how this black-box works, and to even explain the decisions made or how the answer came about, because this information is hidden, unlike classic computer vision algorithms^4–6,17^ in which the result easily interpretable in each computational pipeline phase. Furthermore, neuronal networks have millions of parameters which are usually learned from annotated data, but annotated data is often very expensive to obtain. This problem can become intractable, since a large amount of annotated data is usually required to ensure model generalization.

The method herein proposed attempts to offer a simple solution to many of these complex coiled worm, self-occluded worm, and even fully aggregated, worm problems by means of simple computer vision techniques. This method improves the worm skeleton obtained by considering the width of each *C. elegans*. This new skeleton, unlike the classic one, allows us to divide highly connected bodies which provide information for the pose tracking problem.

## Methods

### *C. elegans* strain and culture conditions

The studied *C. elegans* are wild-type (N2) and CB1370 *daf-2* (e1370), obtained from the Caenorhabditis Genetics Center at the University of Minnesota. Experiments were performed at 20 °C in nematode growth medium (NGM) seeded with *Escherichia coli* strain OP50 (*E. coli*) as standard diet. Synchronised young adult *C. elegans* were obtained from worm eggs incubated at 20 °C in 55 mm-diameter NGM plates. FUdR (0.2 mM) was used to prevent reproduction. All the plates were closed with a lid and fungizone (1 µg/mL) was added to prevent fungal contamination^18^. Plates were cultivated with 10, 15, 30, 60 and 100 worms to obtain a wide variability of cases, aggregation of two *C. elegans* or more, and single rare poses (e.g. coiled shapes, curls, etc.).

### Capture conditions for image sequences

A laboratory operator collects plates from the incubator and places them in the image capture system^17^, where a sequence of 30 images is captured at a frequency of 1 Hz. The time outside the incubator is short, and the ambient temperature is 20 °C to avoid condensation. If condensation appears on plate covers, they are removed by the operator before the image acquisition process. All data was collected with lids, since no condensation problem was detected. *E. coli* OP50 grass was seeded in the centre of plates to prevent worms from going outside the field of view by scaling the edges of plates or near them.

### Illumination of the image capture system

Artificial vision systems depend on various components that work together to acquire, process and analyse images. The lighting system is one of the most important as it allows notable characteristics to be highlighted on images which were, in our case, *C. elegans* processing and the background. The employed technique was an intelligent active backlight illumination that consisted of putting the Petri plate between the lighting system and the camera. As proved in^17^, this lighting method allows constant intensity levels of the background (near the control reference of 48) and the studied *C. elegans* (near the 0 intensity level) to be obtained, which means that it is more robust than standard backlight methods as it narrows the variability of the captured images, and allows fixed segmentation thresholds to be used for all images.

### Image acquisition method

**Figure 1:**
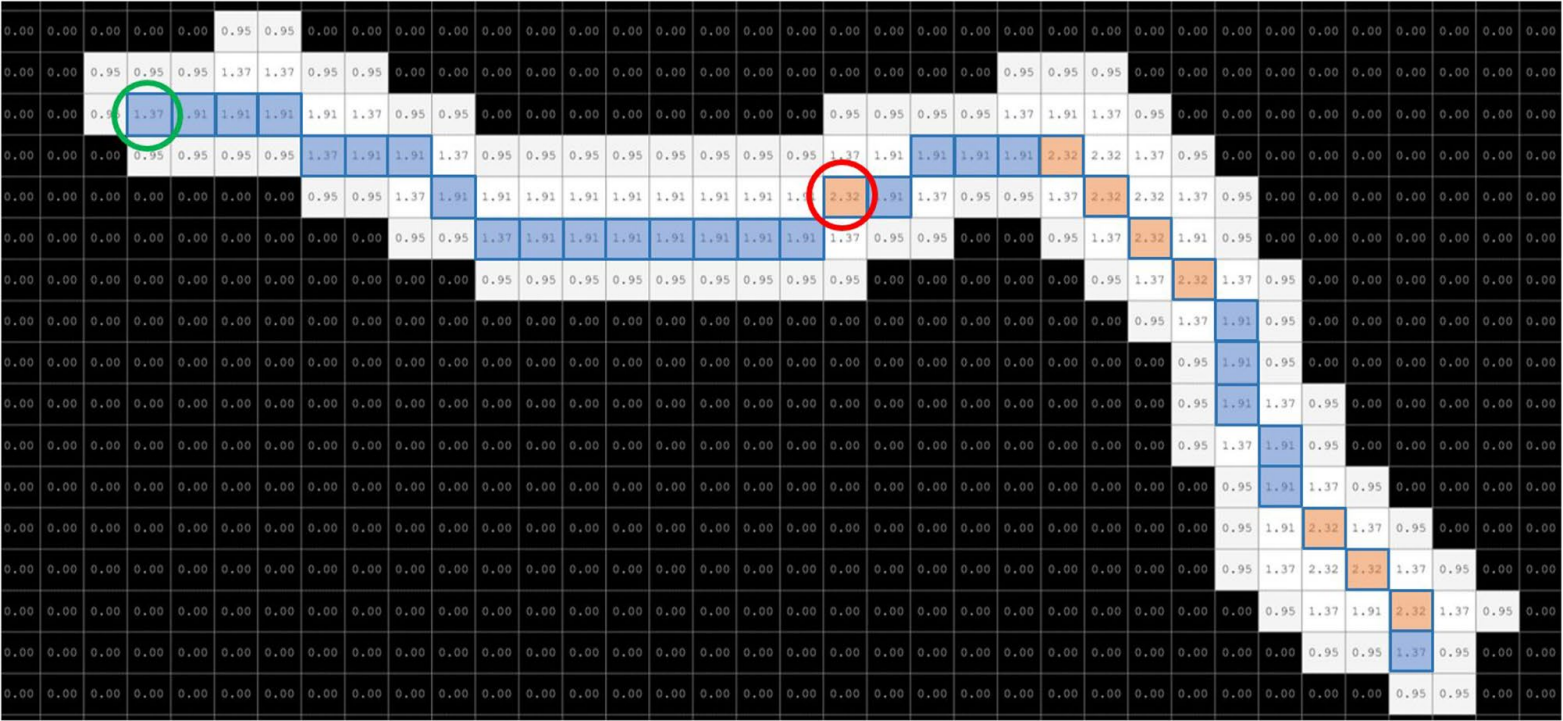
Worm model. Skeleton (blue and orange pixels). Values are the result of the distance transform; Worm model: maximum width value (red circle): 2.32 $\times$ 2 pixels; minimum width value (green circle): 1.37 $\times$ 2 pixels; total length value: 34 pixels.

The image sequence was acquired at a resolution of 1944 $\times$ 1944 pixels and a frequency of 1 Hz (1 image per second) using the system that uses an RGB Raspberry Pi v1.3 camera, OmniVision OV5647, 2592 $\times$ 1944 pixels resolution, 1.4 $\times$ 1.4 $\upmu$m pixel size, 53.50° $\times$ 41.41° field of view, with the 1/4″ optical size and 2.9 focal length, a lighting system based on a 7 Raspberry Pi display at a resolution of 800 $\times$ 480 and 60 fps, 24-bit RGB colour and a Raspberry Pi 3 processing unit. This developed image acquisition system is open hardware. Its assembly procedure, parts and description are detailed in another work^17^. Under these image acquisition conditions, a young adult worm or an adult worm on the plate is projected on image Fig. 1, with a size of 20 or 40 pixels of length and 3 or 5 pixels of width.

### Distance transform function

The distance transform or Euclidean distance map is a tool that transforms an input binary image into a grey-scale image, where each transformed pixel is a decimal value or a brightness value (the values of each pixel in Fig. 1) which corresponds to the Euclidean distance of that pixel to the closest background pixel^19^.

### Classical skeletonisation method

Many classic skeletonisation methods are available as computer vision tools, and each one obtains different skeletons results. We decided to use the Matlab *bwmorph* function as an example of the classic image skeletonisation method. This function’s input parameters include: first, the binary image to skeletonise; second, the operation to be performed on the binary image, in our case ‘thin’; third, the value number of times to apply the operation. If this number is ‘Inf’, the operation will be repeated until the result remains unchanged (coloured pixels in Fig. 1).

### Worm model

The worm model is defined by the following three parameters: the maximum and minimum width values and total length value, obtained by the former methods. To estimate the maximum and minimum width value (maximum value in the red circle in Fig. 1), firstly the transform distance values of the worm image segmentation are obtained. Then the skeleton is obtained with the classic skeletonisation function. On this skeleton, the pixels with the highest and lowest value are found. Total length is the total number of pixels in the skeleton.

### Processing image sequences

As previously mentioned, *C. elegans* are ideal organic models for studying and treating age-related diseases. The most popular assays are lifespan and healthspan. As these assays are usually performed with several synchronised worms per plate, it is important to monitor their behaviour individually or when they aggregate with others. There are some multitrackers available for *C. elegans*^12,20–24^, but they require manually adjusting some parameters like the segmentation threshold. In our case, we used a simple and fully automatic image processing pipeline to take advantage of the intelligent illumination system^25^. The main processing steps to resolve the tracking of worms throughout the imaging sequence are outlined below.

**Figure 2:**
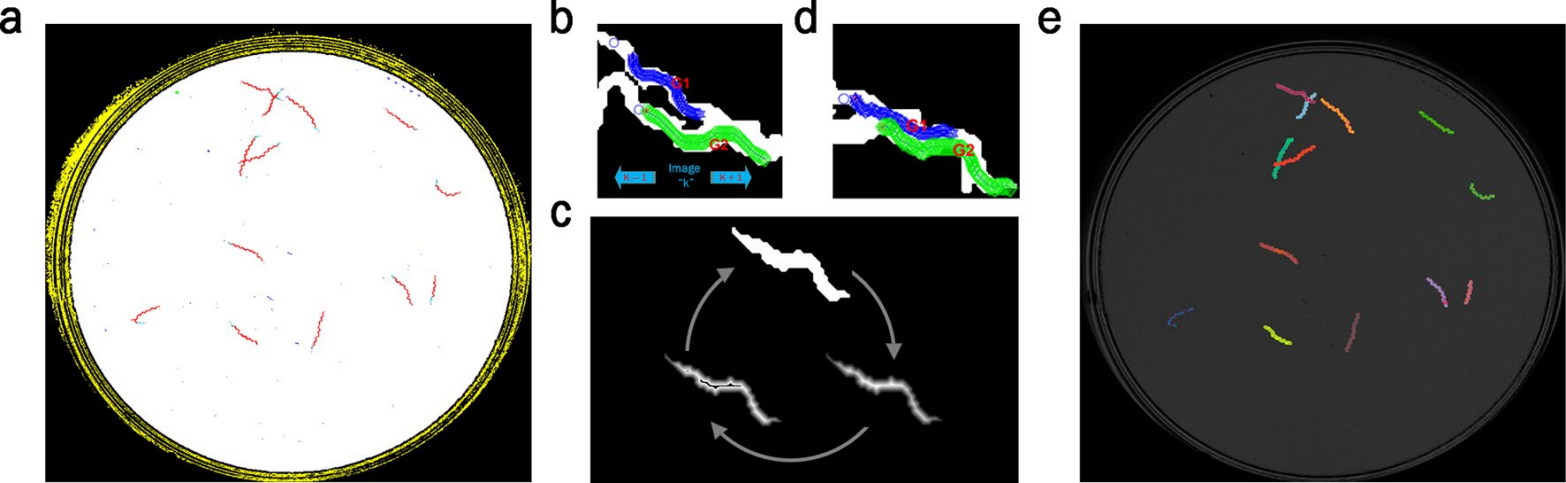
Image processing pipeline. (**a**) Image sequence segmentation result (background in black, region of interest in white, worm tracks in red, noise in blue). (**b**) Worms before aggregation. (**c**) Proposed skeleton technique to obtain separate skeletons. (**d**) Optimisation results (predicted poses). (**e**) *C. elegans* tracking results.

Firstly, the region of interest on the Petri plate is obtained from each image (white circle in Fig. 2a) by applying a simple threshold as all the pixels with an intensity value above 35 on the grey scale belongs to this region of interest. It is worth remarking that work can be done with a fixed segmentation threshold for all these images because the image capture system incorporates a lighting control strategy to keep background levels constant^17^. After this segmentation, an AND operation between all the segmented images in a sequence is run. A close operation (dilating and eroding) is applied to the resulting image to fill in small holes. Finally, the largest connected component is detected as the region of interest.

In parallel to this first step, we also segmented the tracks of *C. elegans* (see Fig. 2a). This process consists of attempting different threshold levels and analysing whether the skeleton of each segmentation corresponds to a minimum expected length. Otherwise it is classified as noise (blue).

Each track is analysed in the second step. For these tracks, the worms in each image are segmented. If it is a multiworm track, images in the sequence are searched for, where all the *C. elegans* are separate and not self-occluded (Fig. 2b). These simple cases allowed us to estimate the worm model, defined by length and width features (Fig. 1). The tracking algorithm starts from one of these simple cases to obtain the worm model before analysing all the sequence both forwardly and backwardly. A new skeleton technique, described in the next section, is proposed to deal with aggregation or self-occluded worms by considering the estimated widths of *C. elegans* (Fig. 2c). Afterwards, an optimisation algorithm is used to obtain the best skeletons (Fig. 2d). Finally, Fig. 2e shows all the tracked worms on a plate.

### Proposed skeletonising method

**Figure 3:**
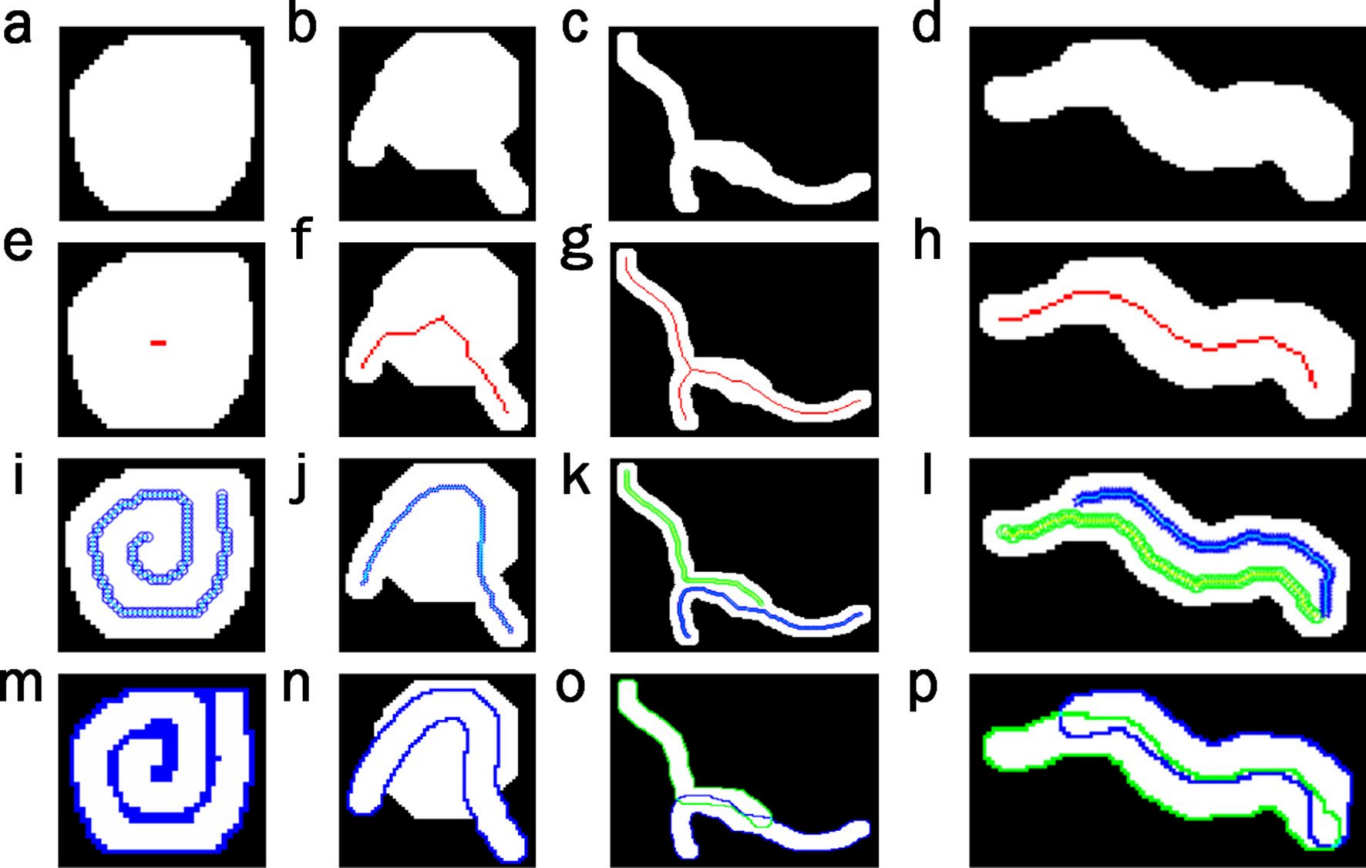
Comparison of skeleton methods. Each of *C. elegans* columns fully wound, *C. elegans* colliding with noise on the plate, *C. elegans* partially in contact, two *C. elegans* traveling in parallel, which correspond to different cases that present problems when tracking is done by a classic skeletisation method with possible loss of identity. (**a**–**d**) Worm segmentation. (**e**–**h**) Skeletons obtained by a classic computer vision method in red. (**i**–**l**) Skeletons obtained by the new proposed method in blue and green. (**m**–**p**) Reconstruction of the *C. elegans* bodies from the skeletons obtained by the proposed method.

Classic image skeletonisation methods, used in computer vision, present major errors in some cases, and may even cause loss of worm identity during tracking. Specifically, these errors occur in those cases in which *C. elegans* are self-occluded or fully rolled (Fig. 3a), when they collide with noise on plates (Fig. 3b), come the head, tail or part of the body comes partially into contact with another worm (Fig. 3c), when two worms travel in parallel for several instants of time (Fig. 3d), etc. The proposed skeleton method obtains a better skeleton in the former cases (Fig. 3i–l) compared to classic methods (Fig. 3e–h). New skeletons are more exact to the centreline of the current position of *C. elegans*, which helps to solve the tracking problem. *C. elegans* bodies can be reconstructed from skeletons by a dilate operation (Fig. 3m–p).

**Figure 4:**
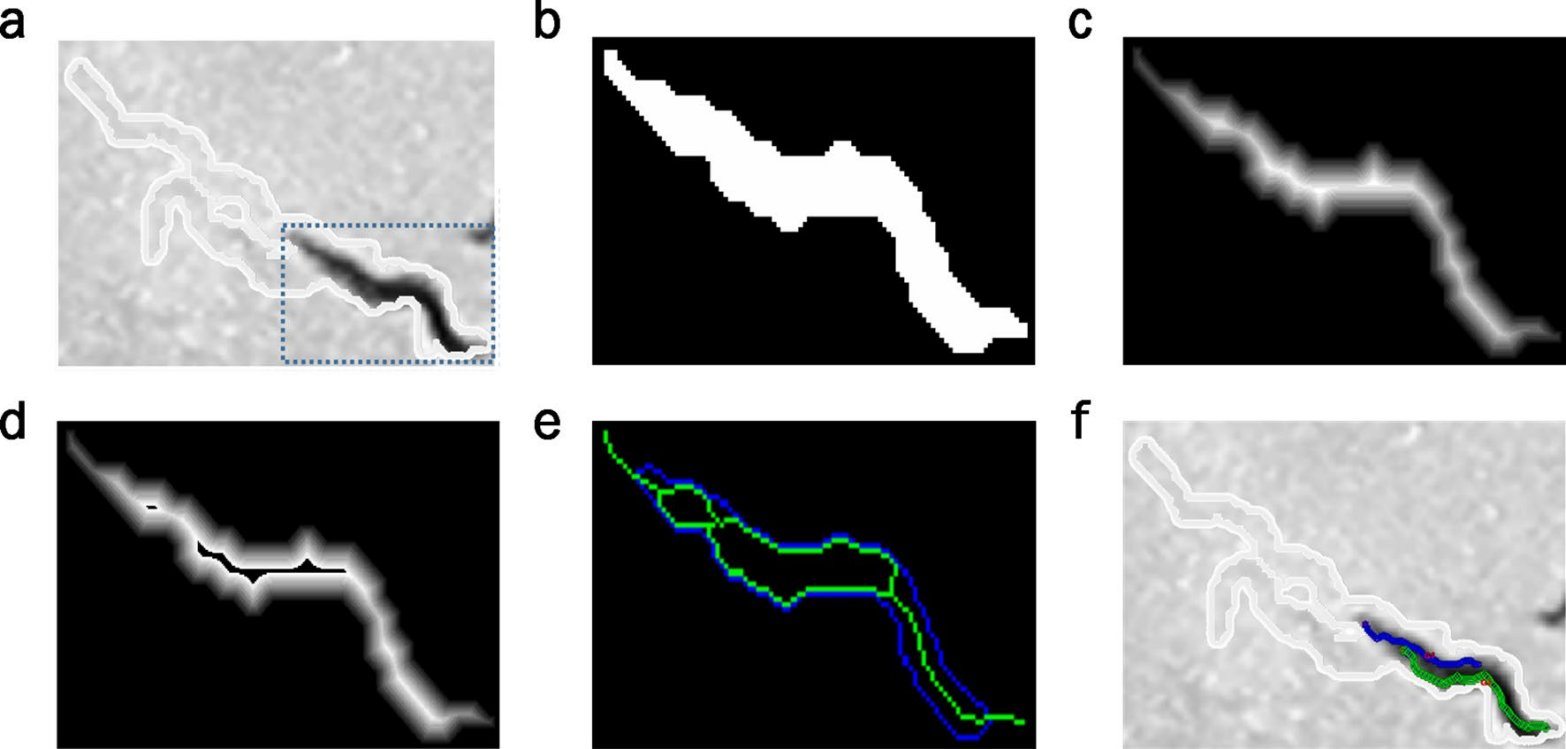
Example of tracks of two connected *C. elegans*. Proposed method steps. (**a**) Cropped subimage containing a current blob. (**b**) Segmentation of the current subimage. (**c**) Distance transform coded by grey intensity levels. (**d**) Background transform by using the maximum width value. (**e**) New skeletons. (**f**) Optimisation result (predicted poses).

The proposed method consists of the following steps. Firstly, a subimage of the bounding box containing the segmented connected region (or blob) is cropped from the current image (Fig. 4a). A blob can be composed of one isolated worm, some connected worms, or others.

Secondly, the subimage is scaled by a factor of 3 (Fig. 4b). Scaling is optional and will be applied, when low-resolution images are used, to more precisely separate aggregated bodies. Thirdly, a distance transform is applied to the connected region (Fig. 4c).

Fourthly, some blob pixels are transformed from the foreground to the background. This transform is known as background transform. The main idea of this method is to transform the pixels in the background to separate connected areas when the blob width is wider than the expected worm width. This means that if there are pixels in the blob with a distance value higher than the maximum width of each model, then these pixels change their values to zero (background value) (Fig. 4d). This process is repeated iteratively until no pixel transforms in the background.

With aggregation, the used width value is the maximum width value estimated in the worm model. If the worm model could not be estimated before aggregation, a default width value can be used. This default value is easy to define because worms are synchronised. This means that all the worms in an experiment are of comparable size (length and width).

Fifthly, a classic skeleton method is applied to the modified blob (after applying the background transform) to obtain separate skeletons (Fig. 4e). It is worth remarking that the proposed method returns the same skeleton as the classic method when no blob pixel is background transformed. In this case, all the distance transform values fall within the expected width.

**Figure 5:**
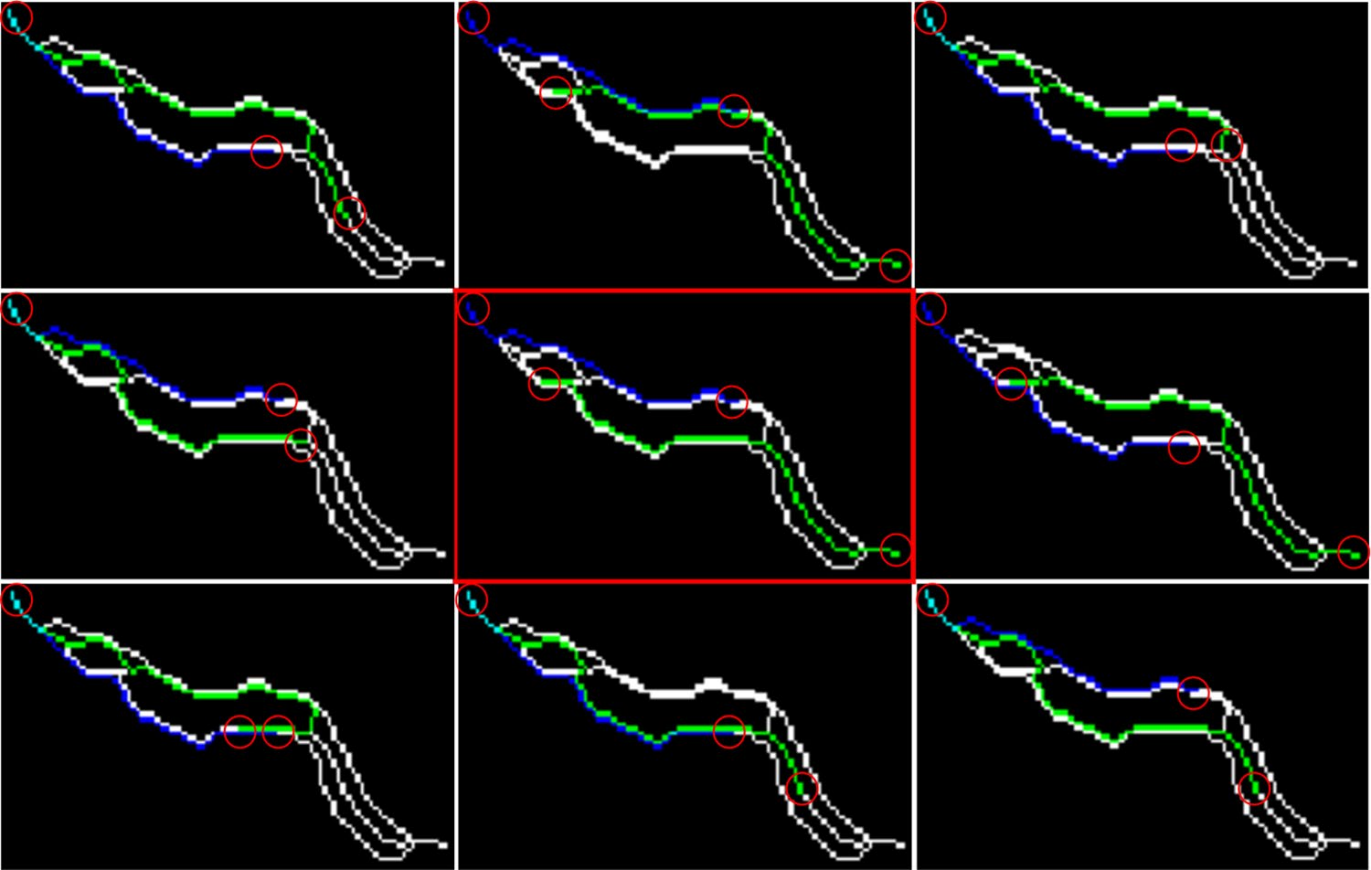
Possible skeleton combinations. Skeleton combinations of two connected worms (blue and green) that will be evaluated by an optimiser to obtain the best possible solution marked by the red square.

Finally, to obtain the best solution (Fig. 4f), all the combinations of poses are analysed (Fig. 5) and the minimum cost is calculated with an optimisation algorithm that evaluates length, width characteristics and the earlier pose state, as described in^25^. All the possible poses are obtained by exploring the continuous paths connecting endpoints and intersections, constrained to the expected lengths. If there are no endpoints and intersections, all the skeleton points overlapping earlier poses are taken. The resulting combinations of poses are filtered to remove similar poses for reducing computational cost. Poses are considered similar when they overlap at least 90 per cent.

### Considerations for rolled *C. elegans* in the proposed skeletonising method

**Figure 6:**
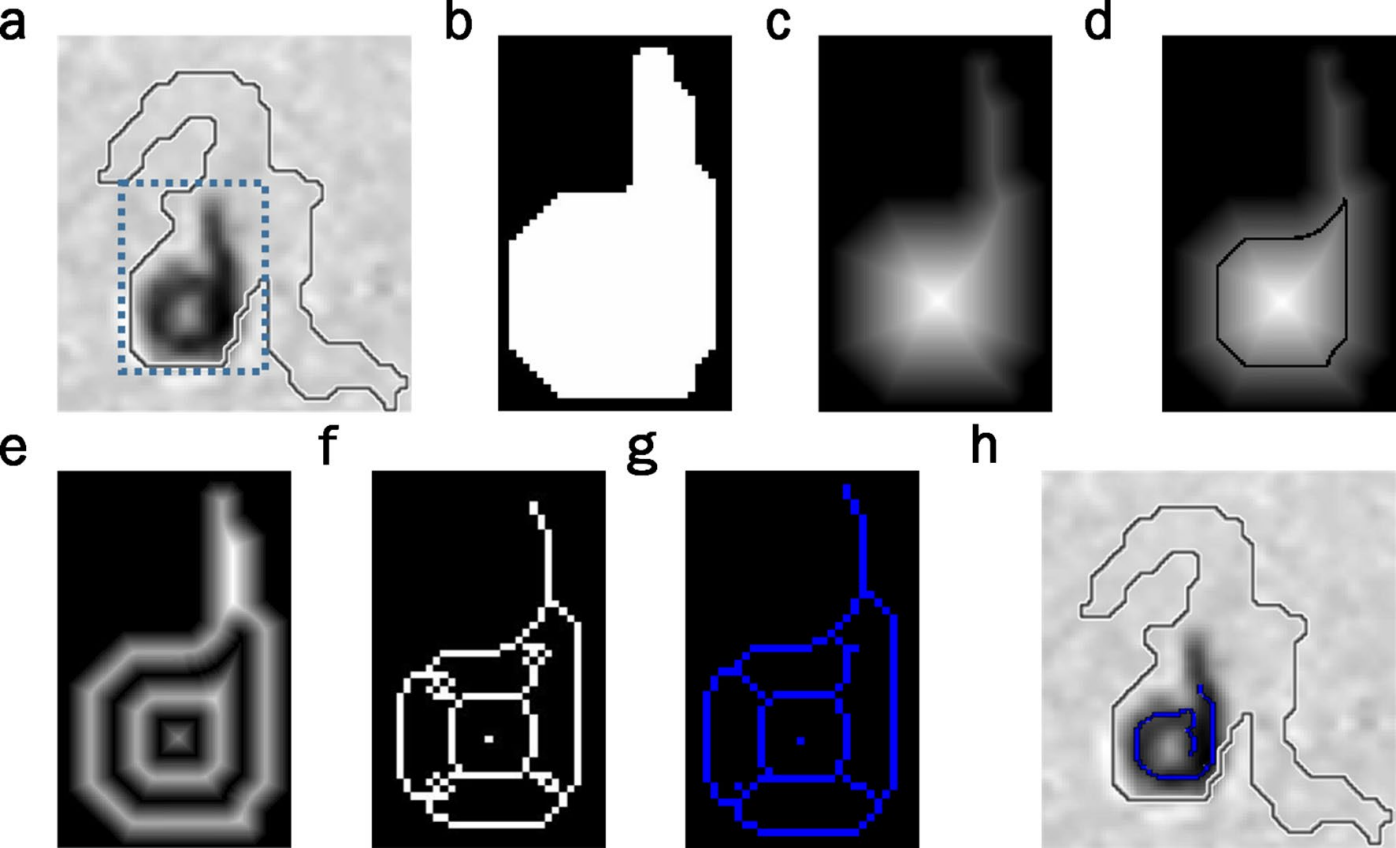
Example of a track of a rolled worm. Proposed method steps. (**a**) Subimage containing the current connected region. (**b**) Segmentation of the current subimage. (**c**) Distance transform. (**d**) Background transform by using the minimum width value. (**e**) Second iteration of the background transform. (**f**) Skeleton with holes. (**g**) Final skeleton. (**h**) Optimisation result.

If a *C. elegans* crawls individually, we proceed similarly with the former method, but the width values to be used in the background transform are the minimum ones in our worm model in order to consider all the coiled shapes, and cases such as coiling completely or partially (Fig. 6a–h). In this example, a second background transform iteration is needed (Fig. 6e). If holes have fewer than 4 pixels in the current resulting skeleton (Fig. 6f), they are filled, and the skeleton is obtained again (Fig. 6g). Filling holes avoids similar skeletons being obtained as possible solutions.

**Figure 7:**
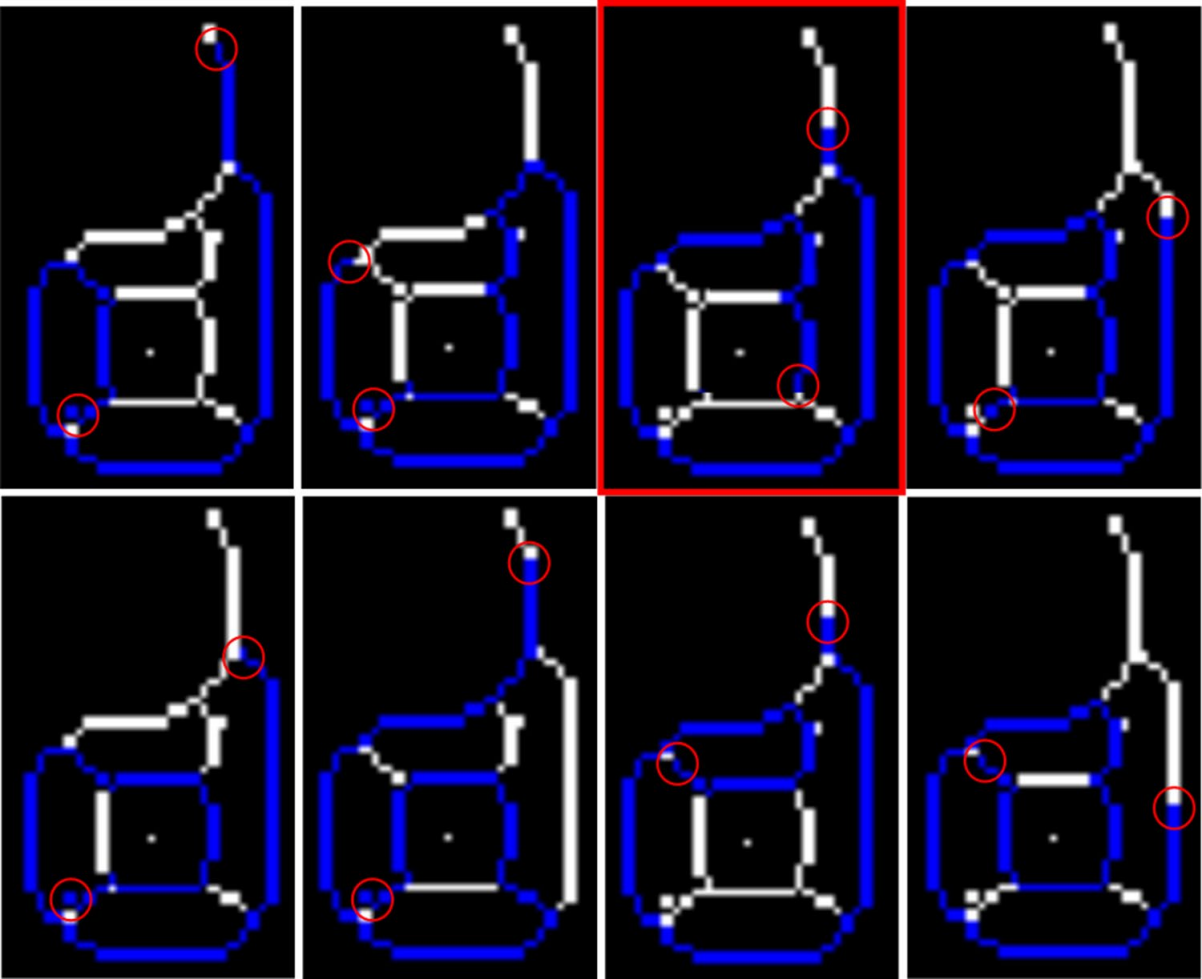
Possible rolled skeleton combinations. Possible rolled skeleton solutions (blue) using the proposed skeletonising method that will be evaluated by an optimiser to obtain the best possible solution marked by the red square.

Finally, to obtain the best solution (Fig. 6h), all the possible solutions extracted from the resulting skeleton are analysed (Fig. 7). The possible skeleton solutions are obtained by exploring any continuous path connecting the endpoints and intersections, constrained to the expected length.

### Validation method

As a reference to compare the results obtained by the different skeletisation methods, ground-true manually labelled skeletons were annotated. This human annotation process was assisted by an application with a friendly interface, designed for helping to select every pixel belonging to the skeleton of each *C. elegans* one by one. This operation was performed for all 205 plates, including the 2196 poses used in the validation phase. The shape of these nematodes was recovered from the annotated skeletons by a dilation operation, applied to the annotated skeletons, with a disk shape of radius that equalled half the width (approx. 1 pixel or 2). This operation returned a shape of 3 or 5 pixels of width. Two pose overlapping indices were used to assess how the methods performed in the validation phase. Both indices were based on the Jaccard coefficient, or junction intersection (IoU). This coefficient measures the degree of precision of the location or detection of objects^26^ and, as its name indicates, it is obtained by dividing the total area of the intersection of elements over the union of these areas^27^ (Eq. 1). For this evaluation, we used the area of the reconstructed bodies from the manually labelled skeletons, the new skeletonising method and the classic skeleton method.

1 $$\begin{aligned} IoU= \dfrac{\sum _{}^{}{P_{w1} \bigcap P_{w2}}}{\sum _{}^{}{P_{w1} \bigcup P_{w2}}}. \end{aligned}$$

**Figure 8:**
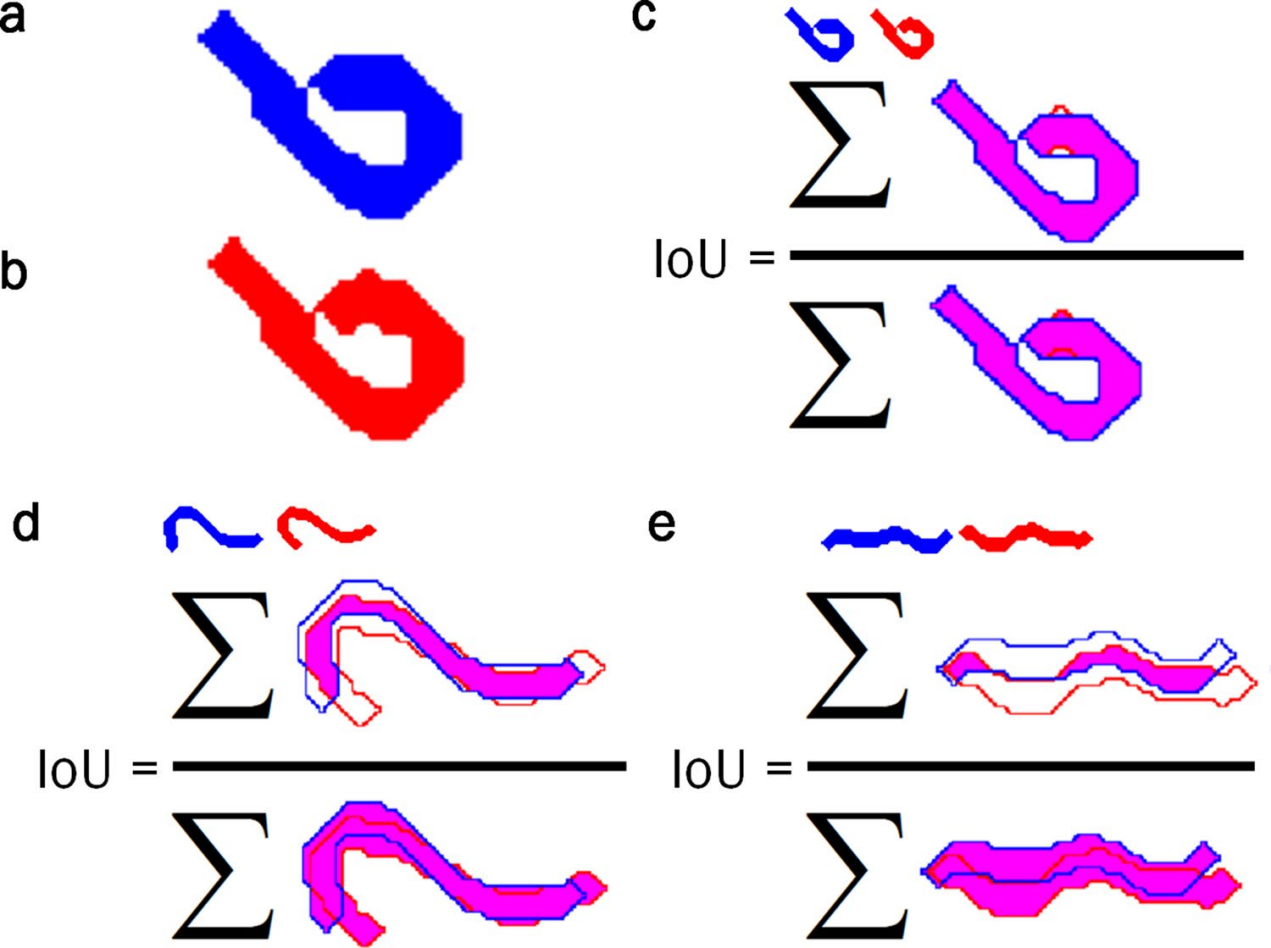
First IoU index. This index evaluates how close the response of the automatic method comes to the reference, and compares both obtained results to determine the improvement of one method compared to the other. The higher the IoU value, the closer the response comes to the reference. The evaluation is performed by reconstructing the skeletons obtained with a radio 2 disk. (**a**) Reconstructed body of the manually labelled skeleton. (**b**) Reconstructed body of the skeleton obtained from the new way to skeletonise. (**c**–**e**) Evaluation of the reconstructed skeletons.

**Figure 9:**
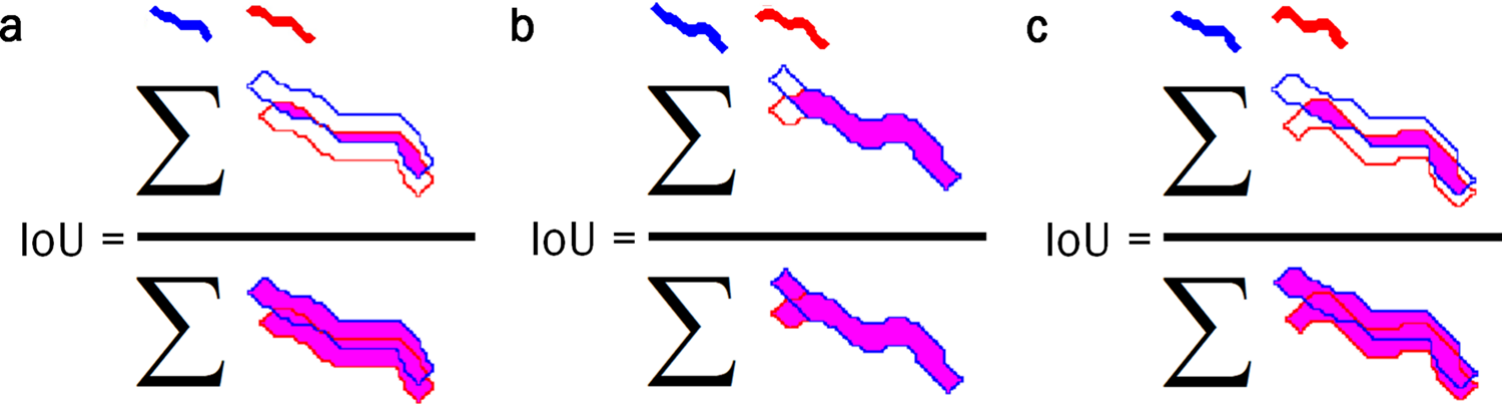
Second IoU index. This index measures how connected two resulting bodies are to one another. The lower the IoU value, the further apart bodies are. Like the earlier method, the evaluation is performed by reconstructing the skeletons obtained with a radio 2 disk. (**a**) Reconstructed bodies from the manually labelled skeletons. (**b**) Reconstructed bodies from the skeletons obtained with the classic method. (**c**) Reconstructed bodies from the skeletons obtained by the proposed method.

The first IoU index was expected to be higher because we compared a predicted pose (Fig. 8b) to an annotated ground-true pose (Fig. 8a) which must overlap (Fig. 8c–e). The results for the shown example are IoU = 0.9784, 0.5667, and 0.2649, respectively. However, the second IoU index was expected to be lower because we compared two predicted poses, in the event of aggregation, which must no overlap one another: Fig. 9a–c. The results for the shown example are IoU = 0.2297, 0.8049, 0.3387 for the reconstructed bodies from the manually labelled skeletons, the skeletons obtained with the classic method and the skeletons obtained by the proposed method, respectively.

The IoU results obtained for each automatic method of 205 plates were analysed by the Wilcoxon Signed Ranks test and T-student statistics with the Statistics and Machine Learning Toolbox of Matlab2018b to obtain the comparison statistics between the two methods and to determine the degree of precision in relation to the reference and the reconstructed body of the manually labelled skeleton (see the complementary material).

### Source code

The programme was developed in Matlab2018b in Windows 10 and works correctly in later versions with the image processing package. Its source code is in GitHub. It is open-source MIT (Massachusetts Institute of Technology) and can be downloaded from the repository at https://github.com/playanaC/Skeletonization.

## Experiments and results

Experiments were performed with 205 plates, of which 193 corresponded to the plates with 10 and 15 worms, 1 plate with 30 worms, 3 plates with 60 worms and 8 plates with 100 worms, which totals 100,000 worm poses. All these data were analysed to obtain all kinds of problematic poses. As proven in^25^ the bigger the number of worms per plate, the more likely them coming into contact with one another. Firstly, 205 plates were analysed, and all the tracks with poses where classic methods fail or have problems identifying each worm were selected in the validation dataset. This dataset contains 2196 poses (2.2% of all poses), including travelling in parallel, partial or total contacts, and coiled shapes. The percentage of contact poses agrees with the contact probabilities in^20,25^. For the analysis, the above-described IoU index1 and 2 were used to compare the manually obtained reconstructed bodies of skeletons (reference), the skeletons obtained with the new method (new Skel) and the skeletons obtained using Matlab function *bwmorph* (classic Skel).

Index1 was employed to evaluate all the mean data for the New skel and classic skel samples. Two tests were used to analyse normality: the Kolmogorov–Smirnov test for large samples (n > 50) and the Shapiro–Wilk test for small samples (n < 50). The *p* value was the criterion adopted to decide suspected normality, which was performed on the difference in both methods. If the *p* value exceeded or equalled the level of significance (5%), the null hypothesis H0 was accepted (data came from normal distribution), otherwise alternative hypothesis H1 was accepted (data did not come from normal distribution). The data from normal distribution were analysed by the Student’s t-test. The data that did not come from normal distribution were analysed by the Wilcoxon signed rank test.

The obtained results showed that our method significantly improved the poses prediction of each *C. elegans* versus the classic method, which allowed us to preserve identity in more cases and to not lose the target during tracking. Table 1 shows the average and normalised results obtained for the different problematic cases, their respective deviations and the improvement achieved using the first IoU index.

**Table 1:** Summary of the comparison of automatic methods with the first IoU index.

Problematic cases	Total tracks	Total pose	Mean IoU skel		SD		Results %
			New	Classical	New	Classical	Improvement
Self-occluded	212	803	0.77	0.76	0.14	0.13	0.91
Noise contact	17	509	0.68	0.66	0.22	0.23	2.07
Partial bodies aggregation	53	828	0.7	0.68	0.18	0.17	2.25
Full bodies aggregation	4	56	0.69	0.47	0.2	0.26	21.5

This table includes the results obtained from the validation dataset and the improvements for each case using the first IoU index. The evaluated values show the percentage of success compared to the reconstructed body of the manually labelled skeleton (see the Supplementary Material for more details).

**Figure 10:**
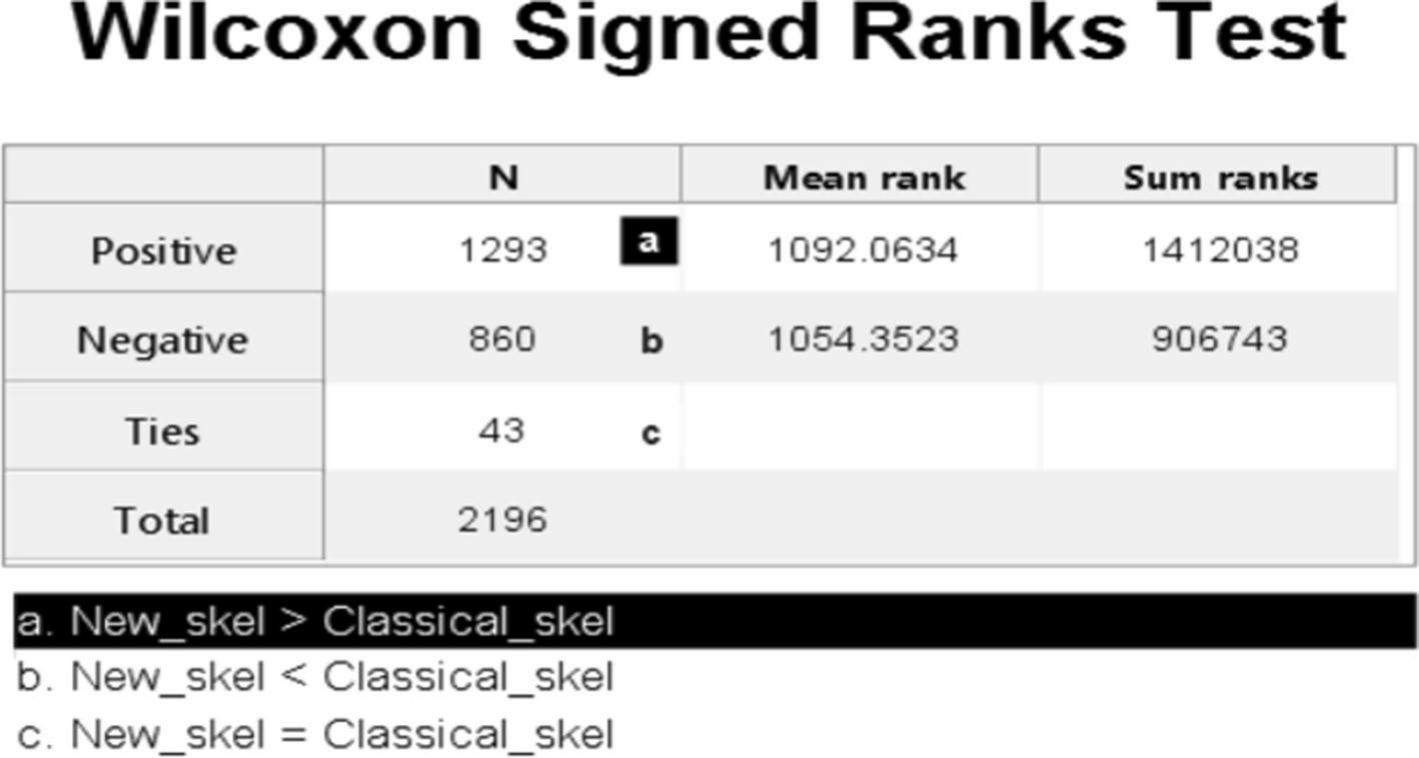
Wilcoxon Signed Ranks Test for the mean-IoU data in Table 1. The graph obtained from the Wilcoxon Signed Ranks test using the Statistics and Machine Learning Toolbox of Matlab2018b. Statistical toolbox indicated a higher positive range value than the negative range value. The *p* value of this test was 1.998E−18 less than the significance value. We can thus conclude that a statistically significant difference appeared between both methods, and the proposed method (New_skel) achieved major improvement compared to the classic method (classical_skel).

The results analysed by the Kolmogorov Smirnov test (N = 2196) showed that the data did not come from normal distribution (*p* value = 4.47E−143). The Wilcoxon Signed Ranks Test was used to analyse data (Fig. 10), which revealed a statistically significant difference between both methods, with a *p* value = 1.99E−18 less than the significance value of 0.05. The proposed method, New_skel, showed a significant improvement (mean = 0.7216, SD = 0.1805, variance = 0.033) over the classic method, classical_skel (mean = 0.6996, SD = 0.1839, variance = 0.034).

Table 2 shows the average and normalised results obtained for connected bodies poses, their respective deviations and the improvement obtained using the second IoU index.

**Table 2:** Summary of the comparison of automatic methods with the second IoU index.

Problematic cases	Total plates	Total pose	Mean IoU skel		SD		Results %
			New	Classical	New	Classical	Improvement
Partial bodies aggregation	53	414	0.07	0.1	0.08	0.11	3.83
Full bodies aggregation	4	28	0.21	0.69	0.2	0.15	48.33

This table offers the results obtained from the connected bodies poses included in the validation dataset using the second IoU index for each problematic case. Values reveal the percentage of how connected they were to one another (See the Supplementary Material for more details).

**Figure 11:**
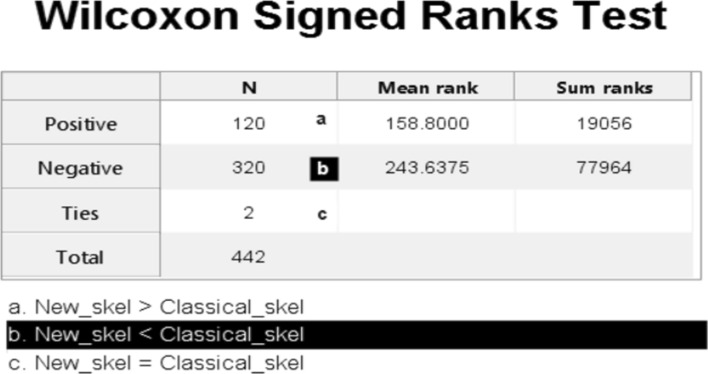
The Wilcoxon Signed Ranks Test for the mean-IoU data in Table 2. The graph obtained from the Wilcoxon Signed Ranks test using the Statistics and Machine Learning Toolbox of Matlab2018b. Statistical toolbox showed a negative ranges value that was higher than the positive ranges value. The *p* value of this test was 2.56E−28 less than the significance value. So we can conclude that a statistically significant difference appeared between both methods and that the proposed method (New_skel) indicated a significant improvement over the classic method (classical_skel).

Index2 was used to evaluate all the mean data for the New skel and Classical skel samples. The results analysed with the Kolmogorov Smirnov test (N=442) showed that the data did not come from normal distribution (*p* value= 7.453E−59). The Wilcoxon Signed Ranks Test was used to analyse data (Fig. 11) and indicated a statistically significant difference between both methods, with a *p* value = 2.56E−28 less than the significance value of 0.05. The proposed method, New_skel, indicated significant improvement (mean = 0.0754, SD = 0.09658, variance = 0. 009) over the classic method, classical_skel (mean = 0.1419, SD = 0.18256, variance = 0.033).

**Figure 12:**
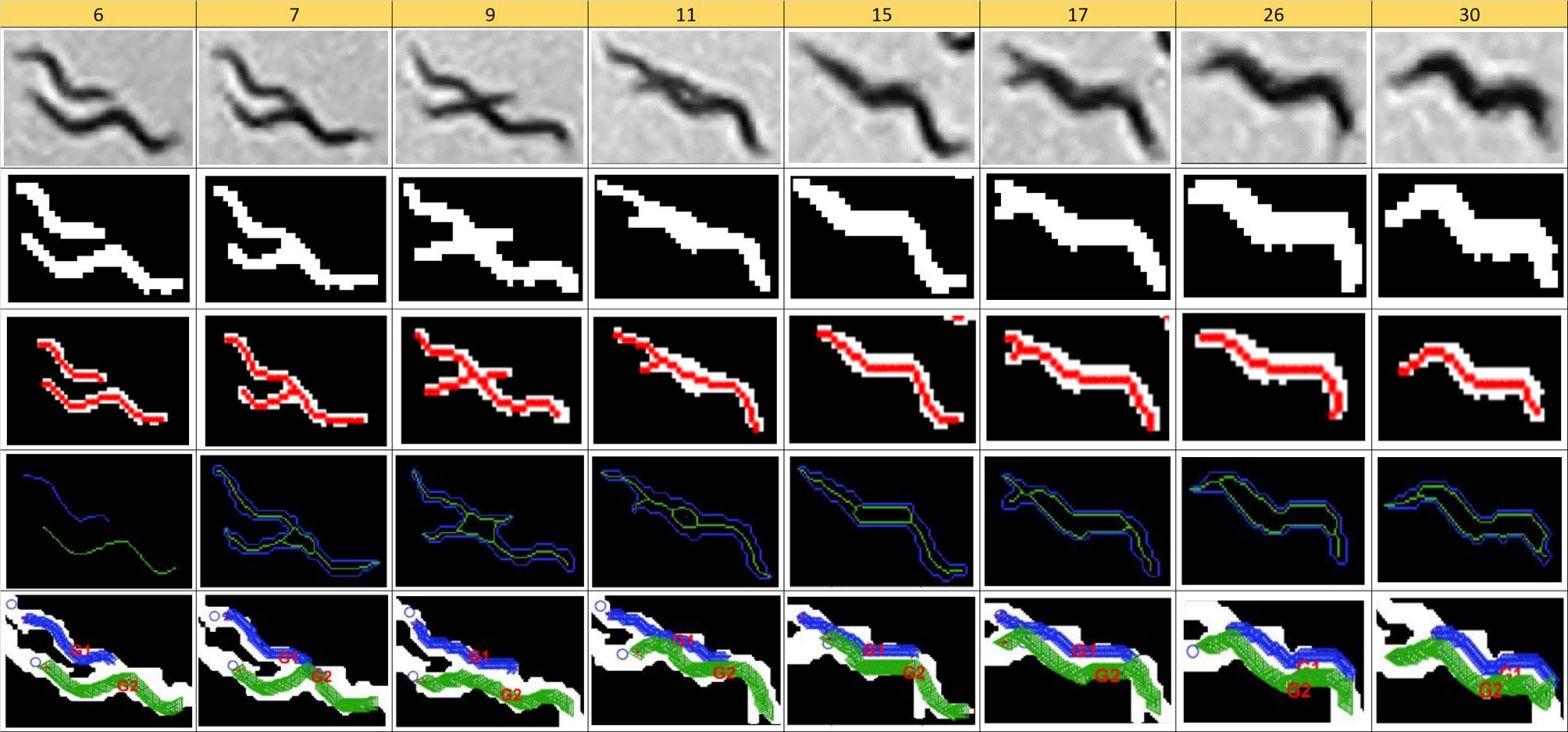
Tracking sequence comparison with both automatic methods. First row: original images; Second row: segmented images; Third row: Classic method skeletons; Fourth row: Proposed method skeletons; Fifth row: Best solutions from new skeletons.

Finally, a sequence of two worms crawling in parallel is shown in Fig. 12. In this case, the best solutions taken from classic skeletons completely overlap, with an undesired high value for the second IoU index. However, the best solutions obtained from the new skeletons overlap less, as expected, for these two worms.

## Discussion

This work presents a new method to obtain better *C. elegans* skeletons to help tracking. This method uses earlier information about the estimated length and widths of each worm before aggregation, self-occlusion, or coiling to change the segmentation image. Skeletonising allows the complex behaviours of these worms to be analysed without losing information on the characteristics of their shape but can, in turn, present problems from reducing information, such as travelling in parallel or rolling up on themselves. All this leads classic methods to fail, and lose identity, location and poses during tracking, as a result of the very little information taken into account. Our method aims to provide a simple solution to this problem by obtaining a new skeleton with information on the width of each worm. This method consists of transforming pixels from the foreground to the background in segmentation whose distance-to-background values are higher than the estimated width. This allows the bodies of each *C. elegans* to be separated to obtain the best possible solution by solving the identity problem of each nematode. A priori, it seems interesting to apply the background transform only to a thin line of pixels. For example, when separating two worms crawling in parallel, it would be interesting to draw a thin background line which cuts the connections between both bodies. However, a problem occurs when attempting to separate these bodies with a thin line (only one pixel width). In this case, skeletons go through diagonal sections of this thin line as the classic skeleton works with 8-connected criteria to connect skeleton pixels. To avoid this problem, a scale factor of 3 is applied to increase image resolution, and then wider lines are used to avoid skeleton connections going through this line. In an individual worm case, such as an entire rolled body, drawing background lines with a width that equals the minimum width model (at least 3 scaled pixels) suffices to solve this problem. However with the aggregation of full bodies, another problem can occur because small discontinuities can appear in the middle of this line. These small holes allow hundreds of similar possible solutions, which incur a high cost to process them all. To solve this problem, we increased the line width to the maximum width model (approx. 9 scaled pixels) to fill these holes and to draw a continuous background line. This extra width reduces pose accuracy, but this reduction is negligible when going back to the low-resolution space. The proposed method returns the same skeleton as the classic method when no blob pixel is transformed from the foreground to the background. This means that all the distance transform function values fall within the expected width. This is the most probable case, where classic methods work well. With 10 or 15 worms per plate (55 mm-diameter), the probability of worm contact is 1%. This probability rises to 2.6%, 4.6%, and 6.6% with 30, 60, and 90 worms per plate, respectively, as proven in^25^. Therefore, classic methods are used with small worm numbers per plate (10, 15 or 30 worms), where the probability of errors is low. However, the proposed method can work with bigger worm numbers per plate and does not diminish tracking performance. Our experiments offer successful results, even with 100 worms per plate. Increasing the number of worms per plate is an interesting issue because it helps to reduce the cost of *C. elegans* experiments. The presented method can be adapted to any multitracker and can be implemented easily in other programming languages given the versatility of the distance transform function, which is found in many image processing toolkits.

## Supplementary Information

Supplementary Infomations.
