# Supplementary material for: Improving skeleton algorithm for helping Caenorhabditis elegans trackers
Source: Sci Rep. 2020 Dec 17;10:22247. doi: 10.1038/s41598-020-79430-8 (PMC7746747; doi:10.1038/s41598-020-79430-8)
Supplement: Supplementary file 1 — Supplementary Infomations. [file 41598_2020_79430_MOESM1_ESM.pdf]

Improving skeleton algorithm for helping *Caenorhabditis elegans* trackers

Pablo E. Layana Castro[1], Joan Carles Puchalt[1], and Antonio-José Sánchez-Salmerón[1,*]

[1]Universitat Politècnica de Valéncia, Instituto de Automática e Informática Industrial, Valencia, Spain
[*]Correspondence to [asanchez@isa.upv.es]

Contents

Summary table IoU index1 **3**

Summary table IoU index2 **4**

Self occluded index1 **6**

Noise contact index1 **7**

Partial bodies aggregation index1 **8**

Full bodies aggregation index1 **9**

Partial bodies aggregation index2 **10**

Full bodies aggregation index2 **11**

Summary table IoU index1

			Mean IoU		Standard Deviation		Results %
Problematic cases	Total tracks	Total pose	New skel	Classical skel	New skel	Classical skel	Improvement
Self-occluded	212	803	0,77	0,76	0,14	0,13	0,91
Noise contact	17	509	0,68	0,66	0,22	0,23	2,07
Partial bodies aggregation	53	828	0,7	0,68	0,18	0,17	2,25
Full bodies aggregation	4	56	0,69	0,47	0,2	0,26	21,5

Supplementary Table 1: Summary of the comparison of automatic methods with the first IoU index. This table shows the results obtained from all the experiments carried out and the improvements for each behavior using the first IoU evaluation method, the evaluated values indicate the percentage of success with respect to the reconstructed body of the skeleton labeled manually. (see experiments and results).

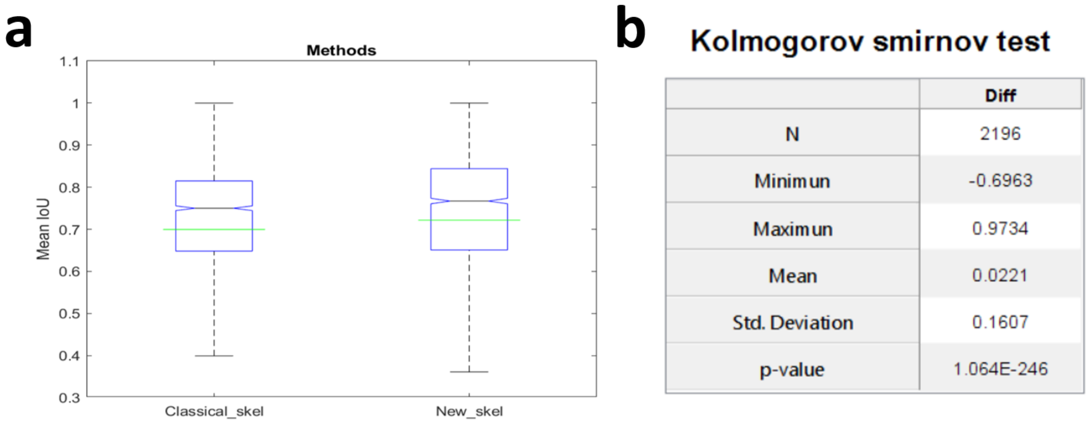

	Diff
N	2196
Minimun	-0.6963
Maximun	0.9734
Mean	0.0221
Std. Deviation	0.1607
p-value	1.064E-246

Supplementary Figure 1: Box plot and normality test of the difference of both methods. (**a**) Box plot, green line indicates the mean in both graphs, and gray line indicates the median. New_skel, N = 2196, mean = 0.7216, median = 0.7671, std. deviation = 0.1805, variance = 0.0326. Classical_skel, N = 2196, mean = 0.6996, median = 0.75, std. deviation = 0.1839, variance = 0.0338. (**b**) Normality test on the difference of methods (new - classical). The p-value obtained was 1.06E-246 less than the significance value of 0.05, so the null hypothesis was rejected and the alternative hypothesis H1 was accepted (data did not come from normal distribution). Once the alternative hypothesis was accepted, Wilcoxon signed ranks test was used to evaluate both methods.

a Wilcoxon Signed Ranks Test

	N		Mean rank	Sum ranks
Positive	1293	a	1092.0634	1412038
Negative	860	b	1054.3523	906743
Ties	43	c		
Total	2196			

a. New_skel > Classical_skel
b. New_skel < Classical_skel
c. New_skel = Classical_skel

b Test Statistics[a]

	New_skel - Classical_skel
z-val	-8.7577[b]
p-value	1.993E-18

a. Wilconxon Signed Ranks Test
b. Based on negative ranks

Supplementary Figure 2: Wilcoxon signed rank test. (**a**) The Wilcoxon signed rank test table shows the difference that exists in 2 related samples through positive, negative and tie ranges. (**b**) P-value obtained with wilcoxon rank test was 1.99E-18 less than the significance value of 0.05, so it was concluded that there was a statistically significant difference between both methods.

Summary table IoU index2

			Mean IoU		Standard Deviation		Results %
Problematic cases	Total tracks	Total pose	New skel	Classical skel	New skel	Classical skel	Improvement
Partial bodies aggregation	53	414	0,07	0,1	0,08	0,11	3,83
Full bodies aggregation	4	28	0,21	0,69	0,2	0,15	48,33

Supplementary Table 2: Summary of the comparison of automatic methods with the second IoU index. This table shows the results obtained from all the experiments carried out and the improvements for each behavior using the second IoU evaluation method, the evaluated values indicate the percentage of how connected they are to each other (see experiments and results).

a

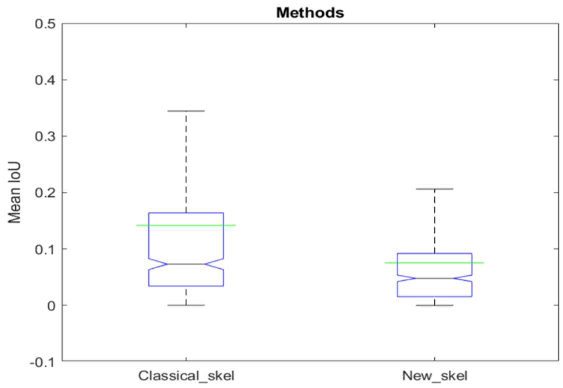

b Kolmogorov smirnov test

	Diff
N	442
Minimun	-0.9678
Maximun	0.5058
Mean	-0.0665
Std. Deviation	0.1574
p-value	1.759E-79

Supplementary Figure 3: Box plot and normality test of the difference of both methods. (**a**) Box plot, green line indicates the mean in both graphs, and gray line indicates the median. New_skel, N = 442, mean = 0.0754, median = 0.0479, std. deviation = 0.0966, variance = 0.0093. Classical_skel, N = 442, mean = 0.1419, median = 0.0732, std. deviation = 0.1826, variance = 0.0333. (**b**) Normality test on the difference of methods (new - classical). The p-value obtained was 1.76E-79 less than the significance value of 0.05, so the null hypothesis was rejected and the alternative hypothesis H1 was accepted (data did not come from normal distribution). Once the alternative hypothesis was accepted, Wilcoxon signed ranks test was used to evaluate both methods.

a Wilcoxon Signed Ranks Test

	N		Mean rank	Sum ranks
Positive	120	a	158.8000	19056
Negative	320	b	243.6375	77964
Ties	2	c		
Total	442			

a. New_skel > Classical_skel
b. New_skel < Classical_skel
c. New_skel = Classical_skel

b Test Statistics[a]

	New_skel - Classical_skel
z-val	-11.0361 [b]
p-value	2.559E-28

a. Wilconxon Signed Ranks Test
b. Based on positive ranks

Supplementary Figure 4: Wilcoxon signed rank test. (**a**) The Wilcoxon signed rank test table shows the difference that exists in 2 related samples through positive, negative and tie ranges. (**b**) P-value obtained with wilcoxon rank test was 2.56E-28 less than the significance value of 0.05, so it was concluded that there was a statistically significant difference between both methods.

Self occluded index1

a

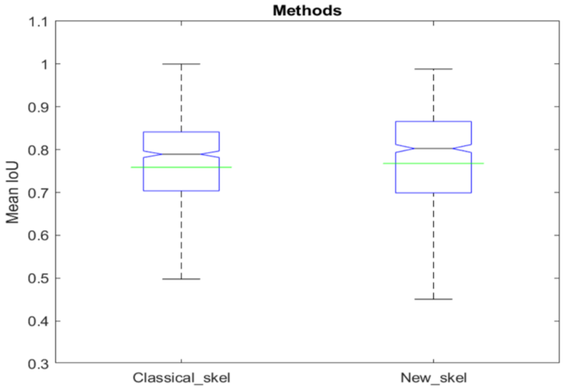

b

Kolmogorov smirnov test

	Diff
N	803
Minimun	-0.6775
Maximun	0.6250
Mean	0.0091
Std. Deviation	0.1130
p-value	1.14E-111

Supplementary Figure 5: Box plot and normality test of the difference of both methods. (**a**) Box plot, green line indicates the mean in both graphs, and gray line indicates the median. New_skel, N = 803, mean = 0.7678, median = 0.8026, std. deviation = 0.1373, variance = 0.0188. Classical_skel, N = 803, mean = 0.7588, median = 0.7892, std. deviation = 0.1290, variance = 0.0166. (**b**) Normality test on the difference of methods (new - classical). The p-value obtained was 1.14E-111 less than the significance value of 0.05, so the null hypothesis was rejected and the alternative hypothesis H1 was accepted (data did not come from normal distribution). Once the alternative hypothesis was accepted, Wilcoxon signed ranks test was used to evaluate both methods.

a **Wilcoxon Signed Ranks Test**

	N		Mean rank	Sum ranks
Positive	451	a	397.1308	179106
Negative	337	b	390.9792	131760
Ties	15	c		
Total	803			

a. New_skel > Classical_skel
b. New_skel < Classical_skel
c. New_skel = Classical_skel

b

Test Statistics[a]

	New_skel - Classical_skel
z-val	-3.7037[b]
p-value	0.0002124

a. Wilconxon Signed Ranks Test
b. Based on negative ranks

Supplementary Figure 6: Wilcoxon signed rank test. (**a**) The Wilcoxon signed rank test table shows the difference that exists in 2 related samples through positive, negative and tie ranges. (**b**) P-value obtained with wilcoxon rank test was 2.12E-4 less than the significance value of 0.05, so it was concluded that there was a statistically significant difference between both methods.

Noise contact index1

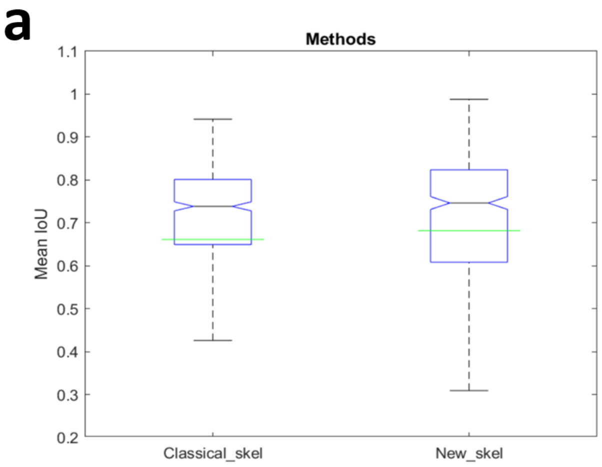

b **Kolmogorov smirnov test**

	Diff
N	509
Minimun	-0.4482
Maximun	0.8235
Mean	0.0207
Std. Deviation	0.1599
p-value	1.036E-62

Supplementary Figure 7: Box plot and normality test of the difference of both methods. (**a**) Box plot, green line indicates the mean in both graphs, and gray line indicates the median. New_skel, N = 509, mean = 0.6818, median = 0.7463, std. deviation = 0.2152, variance = 0.0463. Classical_skel, N = 509, mean = 0.6611, median = 0.7384, std. deviation = 0.2300, variance = 0.0529. (**b**) Normality test on the difference of methods (new - classical). The p-value obtained was 1.04E-62 less than the significance value of 0.05, so the null hypothesis was rejected and the alternative hypothesis H1 was accepted (data did not come from normal distribution). Once the alternative hypothesis was accepted, Wilcoxon signed ranks test was used to evaluate both methods.

a **Wilcoxon Signed Ranks Test**

	N		Mean rank	Sum ranks
Positive	290	a	245.1621	71097
Negative	199	b	244.7638	48708
Ties	20	c		
Total	509			

a. New_skel > Classical_skel
b. New_skel < Classical_skel
c. New_skel = Classical_skel

b **Test Statistics**[a]

	New_skel - Classical_skel
z-val	-3.5807[b]
p-value	0.0003427

a. Wilconxon Signed Ranks Test
b. Based on negative ranks

Supplementary Figure 8: Wilcoxon signed rank test. (**a**) The Wilcoxon signed rank test table shows the difference that exists in 2 related samples through positive, negative and tie ranges. (**b**) P-value obtained with wilcoxon rank test was 3.43E-4 less than the significance value of 0.05, so it was concluded that there was a statistically significant difference between both methods.

Partial bodies aggregation index1

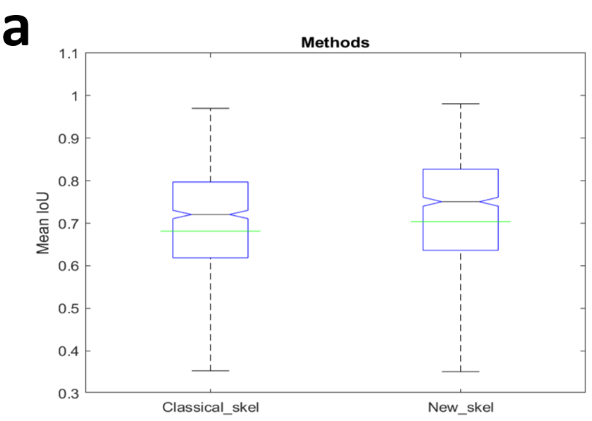

b

Kolmogorov smirnov test

	Diff
N	828
Minimun	-0.6963
Maximun	0.8927
Mean	0.0225
Std. Deviation	0.1790
p-value	6.88244E-85

Supplementary Figure 9: Box plot and normality test of the difference of both methods. (**a**) Box plot, green line indicates the mean in both graphs, and gray line indicates the median. New_skel, N = 828, mean = 0.7038, median = 0.7506, std. deviation = 0.1837, variance = 0.0337. Classical_skel, N = 828, mean = 0.6813, median = 0.7206, std. deviation = 0.1688, variance = 0.0285. (**b**) Normality test on the difference of methods (new - classical). The p-value obtained was 6.88E-85 less than the significance value of 0.05, so the null hypothesis was rejected and the alternative hypothesis H1 was accepted (data did not come from normal distribution). Once the alternative hypothesis was accepted, Wilcoxon signed ranks test was used to evaluate both methods.

a

Wilcoxon Signed Ranks Test

	N		Mean rank	Sum ranks
Positive	508	a	412.8976	209752
Negative	312	b	406.5962	126858
Ties	8	c		
Total	828			

a. New_skel > Classical_skel
b. New_skel < Classical_skel
c. New_skel = Classical_skel

b

Test Statistics[a]

	New_skel - Classical_skel
z-val	-6.1089[b]
p-value	1.00294E-09

a. Wilconxon Signed Ranks Test
b. Based on negative ranks

Supplementary Figure 10: Wilcoxon signed rank test. (**a**) The Wilcoxon signed rank test table shows the difference that exists in 2 related samples through positive, negative and tie ranges. (**b**) P-value obtained with wilcoxon rank test was 1E-9 less than the significance value of 0.05, so it was concluded that there was a statistically significant difference between both methods.

Full bodies aggregation index1

a

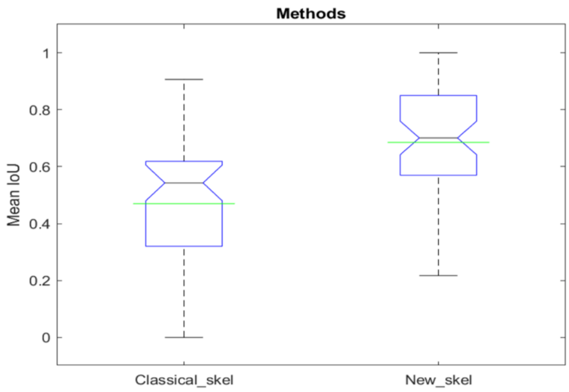

b

Kolmogorov smirnov test

	Diff
N	56
Minimun	-0.2819
Maximun	0.9734
Mean	0.2150
Std. Deviation	0.2927
p-value	0.008028

Supplementary Figure 11: Box plot and normality test of the difference of both methods. (**a**) Box plot, green line indicates the mean in both graphs, and gray line indicates the median. New_skel, N = 56, mean = 0.6852, median = 0.7009, std. deviation = 0.1969, variance = 0.0388. Classical_skel, N = 56, mean = 0.4702, median = 0.5430, std. deviation = 0.2571, variance = 0.0661. (**b**) Normality test on the difference of methods (new - classical). The p-value obtained was 8.03E-3 less than the significance value of 0.05, so the null hypothesis was rejected and the alternative hypothesis H1 was accepted (data did not come from normal distribution). Once the alternative hypothesis was accepted, Wilcoxon signed ranks test was used to evaluate both methods.

a

Wilcoxon Signed Ranks Test

	N		Mean rank	Sum ranks
Positive	44	a	31.9318	1405
Negative	12	b	15.9167	191
Ties	0	c	NaN	
Total	56			

a. New_skel > Classical_skel
b. New_skel < Classical_skel
c. New_skel = Classical_skel

b

Test Statistics[a]

	New_skel - Classical_skel
z-val	-4.9514 [b]
p-value	7.37E-07

a. Wilconxon Signed Ranks Test
b. Based on negative ranks

Supplementary Figure 12: Wilcoxon signed rank test. (**a**) The Wilcoxon signed rank test table shows the difference that exists in 2 related samples through positive, negative and tie ranges. (**b**) P-value obtained with wilcoxon rank test was 7.37E-7 less than the significance value of 0.05, so it was concluded that there was a statistically significant difference between both methods.

Partial bodies aggregation index2

a

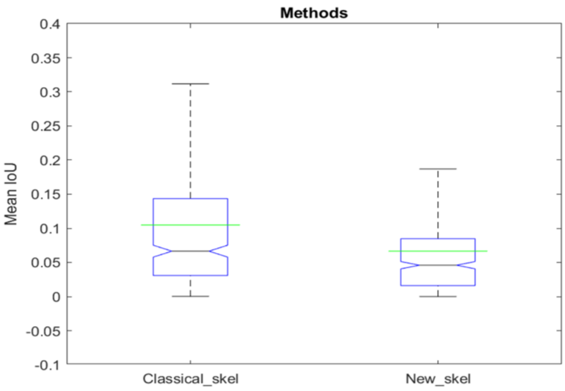

b

Kolmogorov smirnov test

	Diff
N	414
Minimun	-0.4062
Maximun	0.5058
Mean	-0.0383
Std. Deviation	0.0862
p-value	4.014E-74

Supplementary Figure 13: Box plot and normality test of the difference of both methods. (**a**) Box plot, green line indicates the mean in both graphs, and gray line indicates the median. New_skel, N = 414, mean = 0.0665, median = 0.0459, std. deviation = 0.0788, variance = 0.0062. Classical_skel, N = 414, mean = 0.1049, median = 0.0665, std. deviation = 0.1109, variance = 0.0123. (**b**) Normality test on the difference of methods (new - classical). The p-value obtained was 4.01E-74 less than the significance value of 0.05, so the null hypothesis was rejected and the alternative hypothesis H1 was accepted (data did not come from normal distribution). Once the alternative hypothesis was accepted, Wilcoxon signed ranks test was used to evaluate both methods.

a

Wilcoxon Signed Ranks Test

	N		Mean rank	Sum ranks
Positive	117	a	154.8291	18115
Negative	295	b	226.9932	66963
Ties	2	c		
Total	414			

a. New_skel > Classical_skel
b. New_skel < Classical_skel
c. New_skel = Classical_skel

b

Test Statistics[a]

	New_skel - Classical_skel
z-val	-10.0988 [b]
p-value	5.59E-24

a. Wilconxon Signed Ranks Test
b. Based on positive ranks

Supplementary Figure 14: Wilcoxon signed rank test. (**a**) The Wilcoxon signed rank test table shows the difference that exists in 2 related samples through positive, negative and tie ranges. (**b**) P-value obtained with wilcoxon rank test was 5.59E-24 less than the significance value of 0.05, so it was concluded that there was a statistically significant difference between both methods.

Full bodies aggregation index2

a

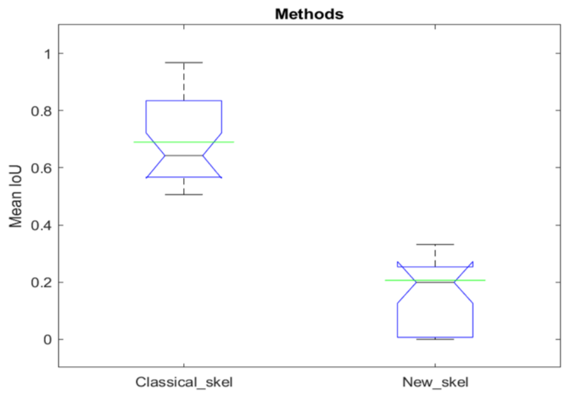

b

Shapiro wilk test

	Diff
N	28
Minimun	-0.9678
Maximun	0.0997
Mean	-0.4833
Std. Deviation	0.3139
p-value	0.01479

Supplementary Figure 15: Box plot and normality test of the difference of both methods. (**a**) Box plot, green line indicates the mean in both graphs, and gray line indicates the median. New_skel, N = 28, mean = 0.2066, median = 0.1993, std. deviation = 0.1958, variance = 0.0383. Classical_skel, N = 28, mean = 0.6899, median = 0.6426, std. deviation = 0.1538, variance = 0.0236. (**b**) Normality test on the difference of methods (new - classical). The p-value obtained was 1.48E-2 less than the significance value of 0.05, so the null hypothesis was accepted (data come from normal distribution). Once the null hypothesis was accepted, t-student test was used to evaluate both methods.

Paired Samples Test

	Mean	Std. Deviation	Lower	Upper	t	df	p-value
New_skel - Classical_skel	-0.4833	0.3139	-0.5776	-0.3889	-10.2718	54	2.624E-14

Supplementary Figure 16: T-student paired samples test. T-student paired samples test table shows the difference that exists in 2 related samples. P-value obtained was 2.62E-14 less than the significance value of 0.05, so it was concluded that there was a statistically significant difference between both methods.
